# Factors Associated with Adherence to the Brazilian Food Guide in Food Service Workers

**DOI:** 10.3390/ijerph20186765

**Published:** 2023-09-15

**Authors:** Lia Silveira Adriano, Brena Barreto Barbosa, Maran Atha Rebelo de Campos, Victoria Maria Ferreira Lima, Eliane Mara Viana Henriques

**Affiliations:** Health Sciences Center, University of Fortaleza, Fortaleza 60811-905, CE, Brazil; brena-barreto@edu.unifor.br (B.B.B.); maran@edu.unifor.br (M.A.R.d.C.); victoriamrflima@edu.unifor.br (V.M.F.L.); elianemara@unifor.br (E.M.V.H.)

**Keywords:** occupational health, food guide, quality of life, occupational groups, food services

## Abstract

The present study aimed to evaluate factors associated with adherence to the Food Guide for the Brazilian Population (GAPB) among food service workers. A cross-sectional study was conducted with 421 employees from 43 food service establishments located in a capital of Brazil. Health and lifestyle data were collected, including the continuous use of medication, smoking, physical activity, and alcohol consumption. An instrument based on the GAPB was utilized, covering the domains of planning, household organization, eating habits, and food choices, according to GAPB recommendations. The findings revealed a direct association between the quality of life scores and the total GAPB adherence score (β = 1.17; *p* < 0.001), as well as the domains of planning (β = 0.53; *p* < 0.001), household organization (β = 0.22; *p* = 0.001), and eating habits (β = 0.38; *p* < 0.001). Adherence to the GAPB among food service workers was strongly linked to their perception of quality of life, in addition to other factors such as gender, age, education, type of work activity, and variables of lifestyle. Therefore, the eating practices of these employees need to be assessed comprehensively, and enhancing their quality of life can encourage proper and healthy eating.

## 1. Introduction

The number of food service workers is growing and the Brazilian food and beverage industry, which includes 37.2 thousand companies, directly generates 1.72 million registered jobs in the sector [1]. These workers, therefore, represent an important segment of the actively working population. Therefore, it is essential to look at their health, in line with the concerns raised by global agencies, such as the World Health Organization (WHO) and the International Labor Organization (ILO), which have expressed a broad consensus that the health and well-being of workers are of paramount importance [2]. Despite the relevance of the segment, few studies have been dedicated to investigating the health and eating habits of this group of workers.

Several studies show that food services can present unfavorable working conditions, such as poor infrastructure, insufficient staff, excessive workload, exposure to noise and high temperatures [3], and high physical and psychological stress [4]. In addition to these conditions, which may vary between companies, the very nature of the work that involves the production of meals means that food handlers have frequent access to food during their work activity, which can influence their relationship with food [5,6]. Often, the foods available are hyper-palatable and rich in fat, sugar, and calories, but poor in minerals and dietary fiber [7,8], and workers are tempted to “try out” or “nibble” between meals. In addition, food handlers working in the restaurant category often do not have the option to go home and eat properly due to long working hours [9].

Given this occupational context, it is important to assess the eating practices of this group of workers from a comprehensive perspective. In 2014, there was important progress for Brazil in that the Ministry of Health launched an update of the Food Guide for the Brazilian Population (GAPB). This edition was developed with an expanded perspective beyond the biological dimension in relation to food and its relationship with health [10]. The recommendations in this edition incorporate social, environmental, and cultural aspects for an adequate, healthy, and sustainable diet [11].

In addition, the recommendations are not based on isolated nutrients, but on food groups, which are considered more coherent to enable understanding between dietary practices and the current epidemiological scenario [12]. The adoption of the NOVA food classification, based on the level of food processing, considers the limitations of the nutritional discourse and starts to incorporate sociocultural and socio-environmental discourses, evoking the pleasure of eating, the diversity of eating habits, and stimulating culinary practices as promoters of healthy eating practices [13].

In 2019, a 24-item scale was published to assess diet according to the Food Guide for the Brazilian Population [14]. From then on, adherence to the recommendations of the national guide was evaluated in certain contexts. Among Brazilian adults, for example, the average score on the scale was 36.4 (8.5) points, with the scores being directly associated with age, as well as being higher among people from the North-Northeast regions compared to those from the Central-West regions [15]. However, there are still few studies that have used this scale and we could not identify studies that evaluated adherence to these recommendations among food service workers. Despite these findings, since the publication of the 2014 guide, the dietary practices of this group of workers according to these recommendations have not yet been investigated. Therefore, this study aimed to evaluate the factors associated with GAPB adherence among food service workers.

## 2. Materials and Methods

### 2.1. Study Design and Subjects

This is a cross-sectional study carried out from May 2019 to May 2023. The city of Fortaleza has a total of 4667 commercial food services, distributed among 2051 snack bars, 1945 restaurants, 383 bakeries and coffee shops, and 288 supermarkets and hypermarkets [16]. The city also boasts a comprehensive educational network, comprising 791 preschools, 951 elementary schools, and 301 high schools [17]. Regarding healthcare infrastructure, Fortaleza has 8 state hospitals [18], in addition to 10 municipal hospitals [19], totaling 2061 institutional healthcare units.

For the sample size calculation, an outcome prevalence of 50% was used due to the uncertainty in the estimate within the studied population. A confidence level of 95% and an error of 5% were considered. Thus, using the G Power software 3.1, the sample was estimated at 385 people. A total of 424 workers were recruited (10% more than the sample calculation to cover possible losses). The companies were recruited for convenience, and all their employees were invited to participate in the study. The invitations were extended in person at the food services by the researchers. Data collection was completed with 421 workers from 43 food services, located in Fortaleza and its metropolitan region in the state of Ceará, Brazil.

This study included employees who work in food services, aged between 18 and 60 years, of both sexes, and who had worked for at least three months in the company. Pregnant women and workers who were unable to answer the questionnaire without the help of others were not included.

### 2.2. Variables

Data collection took place from face-to-face interviews with the workers and through anthropometric assessment. The interview and procedures were carried out by duly trained researchers. The questionnaires were administered to the workers during their work break. The collected variables of the sociodemographic and labor characteristics were age, gender, self-declared race, per capita income, education, current time in employment, type of food service (institutional or commercial), work schedule (6 × 1 day or 12 × 36 h), and work activity (direct food handler, indirect food handler, and professionals who do not handle food). Direct food handlers were those who perform direct handling activities, such as cooks, kitchen assistants, pizza makers, barbecuers, those involved in snack preparation, and confectioners. Indirect handlers were those who serve ready-to-eat food, but do not participate in direct handling, such as waiters, servants, and attendants. Workers who did not handle food were workers in the administrative sectors.

Health and lifestyle data were also collected, such as continuous use of medication; smoking through the Fagerström Tolerance Questionnaire (QTF) [20]; practice of physical activity by the International Physical Activity Questionnaire (IPAQ) [21]; and alcohol use by the Alcohol Use Disorders Identification Test (AUDIT-C) [22]. Subjects were then categorized into smokers and non-smokers; active (at least 150 min of moderate intensity physical activity per week or vigorous intensity of at least 75 min per week) and sedentary [23]; and drinkers (AUDITC Score > 4 for men and >3 for women) and non-drinkers [22].

The quality of life of employees was assessed using the WHOQOL-bref questionnaire (Portuguese version) [24]. This tool consists of 26 questions, which explore physical factors, psychological factors, social relations, environment, and self-assessment of quality of life.

To collect data on workers’ eating practices, the instrument developed by Gabe & Jame (2019) [14] was used. The instrument contains 24 questions covering the domains of planning, household organization, eating habits, and choosing food according to GAPB recommendations. Individuals obtained a general score for eating practices and also a score for each domain.

The instruments used already had Portuguese versions published and validated, and these versions were employed. A pilot study was conducted with employees to identify potential needs for adjustments in the data collection procedure.

The anthropometric data collected were body weight and height. To measure weight, a microdigital electronic scale, Cadence model: BAL150-Bat, with a capacity of 150 Kg and precision of 100 g, was used. Participants were instructed to wear light clothing. Height was measured using an Alturexata^®^ stadiometer (Alturexata Ltda., Belo Horizonte, Brazil) in millimeters. In both situations, the individuals stood up, in a straight position, with their arms relaxed and their heads at a horizontal plane [25].

**Figure 1:**
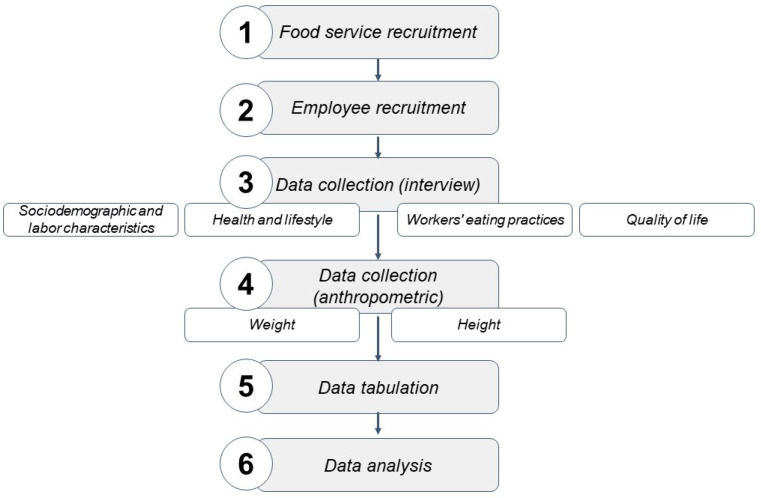
Flowchart with details of the study protocol.

After measuring weight and height, the Body Mass Index (BMI) was calculated, defined by the equation: BMI (Kg/m^2^) = body weight/height^2^ and, subsequently, the workers had their nutritional status categorized into malnutrition, eutrophy, overweight, or with obesity [25]. A flowchart detailing the study protocols is available in Figure 1.

### 2.3. Statistics

Descriptive data were presented as absolute and relative frequency or as mean and standard deviation. The Kolmogorov–Smirnov test was used as a normality test. Continuous variables were compared between genders using Student’s *t* test and categorical variables using Pearson’s chi-squared test. For the association between adherence to the dietary guide (dependent variable) and associated factors (independent variables), a multiple linear regression model was used. The selection of covariates was based on the literature, as well as on the presence of confounding factors and on the collinearity between variables. Effect measurements were presented as beta values and *p* values. Statistical analyses were performed using the Stata program (version 12.0, 2011, StataCorp LP, College Station, TX, USA). *p* < 0.05 was considered significant.

### 2.4. Ethical Aspects

This study was conducted in accordance with the guidelines established in the Declaration of Helsinki and all procedures involving human beings were approved by the Research Ethics Committee of the University of Fortaleza (CAAE nº 40665620.9.0000.5052). Written informed consent was obtained from all research participants.

## 3. Results

A total of 421 adult food service workers were evaluated, of which 50.4% were female. Most workers declared themselves mixed-race or black (78.6%) and were attending or had completed high school (67.0%). Age, self-declared race, and education did not differ between genders (*p* > 0.05) (Table A1).

Most of the evaluated workers worked in commercial food services (79.8%), worked as direct food handlers (56.5%), and worked on a 6 × 1 day scale (93.6%). The type of food service and work activity was similar between male and female (*p* > 0.05). The proportion of males who worked in the 12 × 36 h scale was higher than that of females (*p* < 0.001) (Table A1).

Regarding nutritional status, the mean BMI was 28.0 (5.09) kg/m^2^, being significantly higher in females (*p* = 0.039). Among those evaluated, 69.6% were overweight or with obesity and this proportion also differed between genders (*p* = 0.010). As for the health and lifestyle profile, almost 30% of the workers used medication continuously, with this proportion being higher among females (*p* = 0.001). The prevalence of smokers was 9.3%, sedentary were 33.5%, and drinkers were 50.6%. The proportion of smokers and drinkers was similar between genders, but sedentary lifestyle was more present in males (*p* = 0.040) (Table A2).

In the assessment of quality of life, men had higher scores in the physical and psychological domains and in the self-assessment of quality of life. The total quality of life score was also higher among men (*p* = 0.001) (Table A2). In the assessment of adherence to the GAPB, there was no difference between genders in the overall score and assessment of domains (*p* > 0.05).

In the univariate analysis, adherence to the GAPB was directly associated with age in its overall score (β = 0.20; *p* < 0.001), as well as in the planning domains (β = 0.30; *p* = 0.035) and choice of food (β = 0.12; *p* < 0.001). The scores in the domains of planning (β = 1.06; *p* < 0.031) and eating habits (β = 0.80; *p* = 0.030) were higher in employees of institutional food services compared to commercial ones. The household organization domain had lower scores among indirect food handlers (β = −0.59; *p* = 0.028) compared to direct handlers and higher scores among workers who did not use medication (β = 0.58; *p* = 0.036) compared to those who used. There was an association between BMI and the choice of food domain (β = 0.07; *p* = 0.035) (Table A3).

Smokers had lower overall scores (β = −3.42; *p* = 0.016), as well as in the domains of eating habits (β = −1.09; *p* = 0.035) and choice of food (β = −1.64; *p* = 0.011), compared to non-smokers. The overall score was also lower among drinkers (β = −3.29; *p* < 0.001), as well as in the domains of planning (β = −0.78; *p* = 0.048), eating habits (β = −0.66; *p* = 0.027), and choice of food (β = −1.60; *p* < 0.001), compared to non-drinkers (Table A3).

General quality of life scores were directly associated with general GAPB adherence scores (β = 1.21; *p* < 0.001) and can be seen in the domains of planning (β = 0.53; *p* < 0.001), household organization (β = 0.21; *p* = 0.001), and eating habits (β = 0.35; *p* < 0.001). All quality of life domains were associated with one or more domains of GAPB adherence (Table A3).

In the multivariate analysis, men had lower planning scores (β = −0.84; *p* = 0.031) compared to women. The association with age was maintained only in the overall adherence score (β = 0.20; *p* < 0.001) and in the choice of food domain (β = 0.12; *p* < 0.001). Workers studying or having completed higher education obtained higher overall scores (β = 3.32; *p* = 0.025); this was also reflected in the choice of food domain (β = 1.53; *p* = 0.021). In relation to workers with an elementary level of education and workers studying or having completed secondary school education, there were higher scores on the eating habits domain (β = 0.87; *p* = 0.020) compared to those who had only completed elementary school (Table A4).

The lowest scores in the household organization domain were maintained among indirect food handlers (β = −0.57; *p* = 0.042) compared to direct handlers. Among smokers, the association was maintained only in the choice of food domain (β = −1.36; *p* = 0.027). There was also an inverse association between sitting time during the week and the planning domain (β = −0.003; *p* = 0.037) (Table A4).

There was a direct association between the quality of life scores and the total GAPB adherence score (β = 1.17; *p* < 0.001) and the domains of planning (β = 0.53; *p* < 0.001), household organization (β = 0.22; *p* = 0.001), and eating habits (β = 0.38; *p* < 0.001) (Table A4).

## 4. Discussion

Our study is pioneering in assessing adherence to the Food Guide for the Brazilian Population and its associated factors among food service workers. We found an important association between these workers’ eating practices and their perception of quality of life, as well as an association with other factors, such as sex, age, education, type of work activity, and variables related to lifestyle.

The association of quality of life with food has already been investigated in other studies that evaluated diet through dietary patterns [26,27,28] or the NOVA classification of foods [29,30]. In a systematic review that evaluated the association between eating patterns and quality of life, it was found that “healthy” eating patterns and “Mediterranean” eating patterns are associated with better QoL scores in the physical and mental domains, whereas unhealthy eating patterns and “Western” eating patterns are associated with lower QoL scores [27].

The GAPB, based on the NOVA classification, promotes a varied and balanced diet, emphasizing fresh and minimally processed foods while advising the restriction of added sugars and salt consumption and avoiding ultra-processed foods. It also emphasizes the importance of creating a pleasant eating environment, maintaining a regular eating schedule, and eating with company [10].

As for the studies that used the NOVA classification of foods to assess diet, there are findings that show an inverse association between the consumption of processed and ultra-processed foods and quality of life scores. Among 273 Paraguayan adults, a significant inverse relationship was found between the consumption of processed and ultra-processed foods with quality of life scores assessed by the European Quality of Life-5 Dimensions scale [29]. The association between a high consumption of ultra-processed foods and lower quality of life has also been identified in other groups, such as Brazilian adolescents [31] and adolescents with celiac disease [30]. However, we did not find studies that evaluated this association among adult workers.

In our study, no association was found between the “choice of food” domain and quality of life. This domain encompasses the inclusion of ultra-processed foods in an individual’s normal consumption. This consumption is expressed in the substitution of main meals, the habit of consuming sugary drinks, and in the consumption of snacks between meals [14].

It is important to consider that dietary assessment must transcend the traditional assessment of foods according to their degree of processing or nutrients. Although Brazil has taken a broader look at food in the GAPB, which was published in 2014 [10], the assessment of adherence to these recommendations is much more recent [14] and studies are still scarce. Our study used a validated instrument that goes beyond the assessment of food consumption using the NOVA classification and our findings showed a direct association between quality of life and adherence to the GAPB in general, and includes the domains of planning, household organization, and eating habits. Our data show that, in this population selected in the work context of food services, quality of life is associated with other domains of eating practices.

The habit of smoking was associated with the domain of “choices of food”. We found that scores for the “choices of food” domain were significantly lower among smokers. Studies indicate that smokers generally have a worse quality of diet compared to non-smokers [32,33]. In addition, smokers tend to have a more unsatisfactory health status due to the association of smoking with unhealthy patterns of food consumption, low physical activity, sleep disturbances, and alcohol consumption in adults [34].

The planning domain, in addition to being positively associated with quality of life, was worse among males and was inversely associated with time spent sitting during the week. The planning domain encompasses the acquisition of food, the combination of foods in the form of meals and their consumption, and is related to the dedication undertaken by individuals to their food [14]. In a study that aimed to investigate the association between meal planning and diet quality, women were more likely to plan meals in advance compared to men. Compared with those who do not plan meals, individuals who plan meals are mostly older women, with a higher educational level, higher income, who follow a diet for weight control, and who are physically active [35].

As for the household organization domain, we found that indirect food handlers had worse scores in this aspect when compared to direct food handlers. This domain is associated with the preparation and consumption of meals at home, as well as there being an efficient organization in the supply and preparation of food at home [14]. This organization contributes to time management and helps avoid a lack of ingredients for preparing meals at home. In addition, having more time available is related to a greater number of meals cooked at home, an increase in the consumption of servings of fruits and vegetables, and a reduction in sodium consumption [36], which can allow people to prepare more diversified recipes [35]. In a study with food handlers in Brazil (direct and indirect handlers not differentiated), it was found that, due to long working hours and low income, these professionals tend to have their meals in the workplace, with little variety [9], which may contribute to lower scores in the household organization. Regarding the findings of our study, we assume that, with direct food handlers, the culinary skills resulting from their work activity can contribute to better scores in this domain compared to workers who do not handle food directly. Moreover, culinary skills are commended in the GAPB, which can contribute to better scores [10].

We also found that the eating habits domain, in addition to being associated with quality of life, was higher among employees with secondary education, compared to elementary school. This domain addresses how food consumption and meals occur, specifically in relation to regularity, attention, and characteristics of the environment [14]. The time and attention dedicated to the act of eating, the environment in which one eats, the presence of distractions, and the sharing of meals and related tasks are elements that influence eating habits [15]. Inadequate eating habits tend to favor the consumption of ultra-processed foods [37] and were associated with lower quality of life [38]. On the other hand, positive eating habits, such as shared meals, have been associated with better nutritional and metabolic health, as well as a better quality of life [39]. In a study that investigated the relationship between diet quality and eating habits of adults, it was observed that higher educational levels are inversely related to the practice of skipping meals and watching television during meals [40].

It is important to highlight that, despite not finding an association between nutritional status and adherence to the GAPB, it was observed that 69.6% of food handlers were overweight or with obesity. In Brazil, being overweight among food handlers is common [9,41,42]. In 2021, the prevalence of overweight and obesity among adults in the country was 57.2% [43]. The adoption of food planning and the organization of the home environment in order to favor healthy choices and eating habits can prevent impulsive eating behaviors, promote a balanced diet, and reduce the risk of developing excess weight. These strategies are important elements for maintaining an adequate body weight and can contribute to a healthy lifestyle [44].

This study has some limitations, since it is a cross-sectional study, which prevents establishing causal relationships. In our study, quality of life was associated with the domains of planning, household organization, and eating habits. However, we cannot exclude reverse causality. Planning food, organizing the home environment, and having better eating habits can result in a better quality of life. Furthermore, it is important to mention that the participation of food handlers in this study was voluntary and the refusal of some participants may have introduced a systematic bias in the results. In regard to strengths of this study, we highlight the sample size and the assessment of their diet having undergone a broader view, as this study was carried out with methodological rigor using the processes of face-to-face data collection, tabulation, and data analysis.

## 5. Conclusions

We can conclude that adherence to the GAPB among food service workers is strongly related to their perception of quality of life, in addition to other factors such as gender, age, education, type of work activity, and variables related to lifestyle. It is worth mentioning that the recommendations of the Brazilian Food Guide have already served as a model for other food guides around the world. These results suggest that quality of life and socioeconomic factors can positively impact the adoption of healthy eating practices. Therefore, the eating practices of these employees need to be assessed comprehensively, and enhancing their quality of life can encourage proper and healthy eating.

The study results also provide a strong foundation for the development of targeted interventions aimed at improving the dietary habits and quality of life of food service workers. This may encompass awareness programs, nutritional education, access to healthy food in the workplace, and wellness policies.

## Data Availability

The data presented in this study are available in the article.

## Appendix A

**Table A1:** Socio-demographic and labor characterization of food service workers.

Socio-Demographic and Labor Characterization Variables	Female	Male	Total	*p*
	*n* = 212	*n* = 209	*n* = 421	
	**Mean (SD)**	**Mean (SD)**	**Mean (SD)**	
**Age, years**	34.01 (10.00)	34.07 (11.26)	34.04 (10.73)	0.956 *
	***n* (%)**	***n* (%)**	***n* (%)**	
**Self-declared race**				0.293 **
White	30 (14.20)	40 (19.10)	70 (16.60)	
Mixed-race/black	170 (80.20)	161 (77.00)	331 (78.60)	
Others	12 (5.70)	8 (3.80)	20 (4.80)	
**Education**				0.418 **
Elementary unfinished/completed	51 (24.10)	40 (19.10)	91 (21.60)	
Secondary unfinished/completed	136 (64.20)	146 (69.90)	282 (67.00)	
Higher unfinished/completed	25 (11.80)	23 (11.00)	48 (11.40)	
**Type of food service**				
Institutional	43 (20.30)	42 (20.10)	85 (20.20)	0.962 **
Commercial	169 (79.70)	167 (79.90)	336 (79.80)	
**Work activity**				
Direct handling of food	116 (54.70)	122 (58.40)	238 (56.50)	
Indirect handling of food	69 (32.50)	74 (35.40)	143 (34.00)	0.074 **
No handling of food	27 (12.70)	13 (6.20)	40 (9.50)	
**Work schedule**				
6 × 1 days	209 (98.60)	185 (88.50)	394 (93.60)	<0.001 **
12 × 36 h	3 (1.40)	24 (11.50)	27 (6.40)	

Source: elaborated by the authors. SD: standard deviation. * Student’s *t* test. ** Pearson’s chi-squared test.

**Table A2:** Health and lifestyle profile of food service workers.

Health and Lifestyle Data	Female	Male	Total	*p*
	*n* = 212	*n* = 209	*n* = 421	
	**Mean (SD)**	**Mean (SD)**	**Mean (SD)**	
**BMI, kg/m²**	28.47 (5.50)	27.44 (4.60)	27.96 (5.09)	0.039 *
	***n* (%)**	***n* (%)**	***n* (%)**	
**BMI (classification)**				0.010 **
Malnutrition	6 (2.80)	1 (0.50)	7 (1.70)	
Eutrophy	51 (24.10)	70 (33.50)	121 (28.70)	
Overweight	78 (36.80)	85 (40.70)	163 (38.70)	
With obesity	77 (36.30)	53 (25.40)	130 (30.90)	
**Continuous use of medication**				0.001 **
Yes	75 (35.40)	44 (21.10)	119 (28.30)	
No	137 (64.60)	165 (78.90)	302 (71.70)	
**Smoker**				
Yes	17 (8.00)	22 (10.50)	39 (9.30)	0.404 **
No	195 (92.00)	187 (89.50)	382 (90.70)	
**Sedentarism**				0.040 **
Active	151 (71.20)	129 (61.70)	280 (66.50)	
Sedentary	61 (28.80)	80 (38.30)	141 (33.50)	
**Time spent sitting in one day during the week (minutes)**	155.59 (120.91)	147.52 (147.86)	151.60 (134.80)	0.541 *
**Time spent sitting in one day during the weekend (minutes)**	213.37 (168.97)	216.26 (195.85)	214.80 (182.50)	0.872 *
**Alcohol drinking**				0.559 **
Non-drinker	108 (50.90)	100 (47.80)	208 (49.40)	
Drinker	104 (49.10)	109 (52.20)	213 (50.60)	
**Quality of life (score)**				
Physical domain	15.14 (2.43)	15.89 (2.07)	15.52 (2.28)	0.001 *
Psychological domain	15.39 (2.49)	16.17 (1.20)	15.78 (2.30)	0.001 *
Social relations domain	15.28 (3.27)	15.67 (2.64)	15.47 (2.98)	0.179 *
Environmental domain	13.46 (2.54)	13.87 (2.12)	13.67 (2.34)	0.075 *
Self-evaluation	14.00 (3.10)	14.62 (2.89)	14.31 (3.01)	0.035 *
Total score	14.61 (2.01)	15.21 (1.67)	14.91 (1.87)	0.001 *
**Adherence scores to the Food Guide for the Brazilian Population**				
Planning	9.25 (4.30)	8.69 (3.80)	8.97 (4.06)	0.154 *
Household organization	7.29 (2.45)	7.06 (2.67)	7.17 (2.56)	0.356 *
Eating habits	11.64 (2.91)	11.73 (3.25)	11.68 (3.08)	0.752 *
Choice of food	10.99 (4.04)	11.17 (3.60)	11.08 (3.82)	0.636 *
Total score	35.42 (8.49)	37.94 (8.51)	35.18 (8.50)	0.561 *

Source: elaborated by the authors. SD: standard deviation. BMI: body mass index. * Student *t* test. ** Pearson’s chi-square test.

**Table A3:** Univariate analyzes of factors associated with adherence to the Food Guide for the Brazilian Population.

Variables	General Score β (*p*) *	Planning β (*p*) *	Household Organization β (*p*) *	Eating Habits β (*p*) *	Choice of Food β (*p*) *
**Sex**					
Female	Reference	Reference	Reference	Reference	Reference
Male	−0.48 (0.561)	−0.56 (0.154)	−0.23 (0.356)	0.09 (0.752)	0.17 (0.636)
**Age, years**	0.20 (<0.001)	0.03 (0.035)	0.01 (0.179)	0.01 (0.372)	0.12 (<0.001)
**Self-declared race**					
White	Reference	Reference	Reference	Reference	Reference
Mixed-race/black	−0.90 (0.416)	−0.58 (0.275)	−0.34 (0.310)	0.38 (0.340)	−0.17 (0.724)
Others	2,00 (0.353)	0.7 (0.497)	−0.30 (0.637)	1.07 (0.171)	0.97 (0.313)
Education					
Elementary completed/unfinished	Reference	Reference	Reference	Reference	Reference
Secondary completed/unfinished	−0.48 (0.634)	−0.16 (0.732)	−0.007 (0.981)	0.62 (0.091)	−0.83 (0.068)
Higher completed/unfinished	1.47 (0.330)	0.93 (0.195)	−0.24 (0.587)	0.05 (0.925)	062 (0.358)
**Type of Food Service**					
Commercial	Reference	Reference	Reference	Reference	Reference
Institutional	1.33 (0.196)	1.06 (0.031)	0.40 (0.196)	0.80 (0.030)	−0.58 (0.208)
**Work activity**					
Direct handling of food	Reference	Reference	Reference	Reference	Reference
Indirect handling of food	−1.10 (0.219)	−0.51 (0.227)	−0.59 (0.028)	0.36 (0.260)	−0.25 (0.528)
No handling of food	−0.06 (0.965)	1.04 (0.130)	−0.33 (0.446)	−0.70 (0.183)	−0.07 (0.912)
**Work schedule**					
6 × 1 days	Reference	Reference	Reference	Reference	Reference
12 × 36 h	−1.22 (0.470)	−0.68 (0.398)	−0.62 (0.223)	−0.29 (0.630)	0.15 (0.840)
**Use of medication**					
Yes	Referência	Referência	Referência	Referência	Referência
No	−0.09 (0.914)	−0.07 (0.871)	0.58 (0.036)	0.33 (0.319)	−0.66 (0.109)
**BMI, kg/m²**	0.06 (0.397)	−0.02 (0.536)	0.01 (0.585)	−0.006 (0.822)	0.07 (0.035)
**Smoking**					
Non-smoker	Referência	Referência	Referência	Referência	Referência
Smoker	−3.42 (0.016)	−1.04 (0.126)	0.006 (0.988)	−1.09 (0.035)	−1.64 (0.011)
**Alcoholic drinking**					
Non-drinker	Referência	Referência	Referência	Referência	Referência
Drinker	−3.29 (<0.001)	−0.78 (0.048)	−0.15 (0.545)	−0.66 (0.027)	−1.60 (<0.001)
**Physical activity**					
Sedentary	Referência	Referência	Referência	Referência	Referência
Active	0.09 (0.918)	0.22 (0.589)	0.01 (0.953)	−0.04 (0.879)	−0.10 (0.788)
**Time sitting during the week**	−0.007 (0.022)	−0.002 (0.076)	−0.001 (0.161)	−0.001 (0.107)	−0.001 (0.300)
**Time sitting at the weekend**	−0.002 (0.293)	−0.0006 (0.551)	0.0002 (0.758)	0.0003 (0.634)	−0.001 (0.097)
**General quality of life**	1.21 (<0.001)	0.53 (<0.001)	0.21 (0.001)	0.35 (<0.001)	0.19 (0.055)
Physical domain	0.83 (<0.001)	0.29 (0.001)	0.15 (0.004)	0.30 (<0.001)	0.14 (0.080)
Psychological domain	0.76 (<0.001)	0.35 (<0.001)	0.14 (0.009)	0.18 (0.004)	0.10 (0.189)
Social relations domain	0.21 (0.132)	0.15 (0.018)	0.06 (0.128)	0.04 (0.357)	−0.001 (0.751)
Environmental domain	0.85 (<0.001)	0.40 (<0.001)	0.12 (0.017)	0.23 (<0.001)	0.15 (0.054)
Self-evaluation	0.65 (<0.001)	0.25 (<0.001)	0.10 (0.015)	0.21 (<0.001)	0.14 (0.020)

Source: elaborated by the authors. * Univariate linear regression. Values expressed in beta and *p* value. BMI: Body Mass Index.

**Table A4:** Multiple analyses of factors associated with adherence to the Food Guide for the Brazilian Population.

Variables	General Score β (*p*) *	Planning β (*p*) *	Household Organization β (*p*) *	Eating Habits β (*p*) *	Choice of Food β (*p*) *
**Sex**					
Female	Reference	Reference	Reference	Reference	Reference
Male	−1.26 (0.117)	−0.84 (0.031)	−0.44 (0.085)	−0.31 (0.299)	0.22 (0.530)
**Age, years**	0.20 (<0.001)	0.03 (0.105)	0.01 (0.559)	0.01 (0.391)	0.12 (<0.001)
**Education**					
Elementarycompleted/unfinished	Reference	Reference	Reference	Reference	Reference
Secondary completed/unfinished	1.44 (0.149)	0.32 (0.511)	0.24 (0.455)	0.87 (0.020)	0.02 (0.949)
Higher completed/unfinished	3.32 (0.025)	1.23 (0.089)	−0.07 (0.875)	0.42 (0.441)	1.53 (0.021)
**Type of Food Service**					
Commercial	Reference	Reference	Reference	Reference	Reference
Institutional	0.71 (0.459)	0.81 (0.085)	0.34 (0.276)	0.60 (0.095)	−0.74 (0.085)
**Work activity**					
Direct handling of food	Reference	Reference	Reference	Reference	Reference
Indirect handling of food	−0.55 (0.526)	−0.47 (0.266)	−0.57 (0.042)	0.45 (0.166)	0.09 (0.817)
No handling of food	−0.16 (0.909)	0.48 (0.491)	−0.26 (0.569)	−0.60 (0.267)	0.17 (0.785)
**Smoking**					
Non-smoker	Reference	Reference	Reference	Reference	Reference
Smoker	−2.64 (0.057)	−0.90 (0.181)	0.14 (0.741)	−0.76 (0.139)	−1.36 (0.027)
**Time sitting during the week**	−0.004 (0.134)	−0.003 (0.037)	−0.0009 (0.347)	−0.001 (0.339)	0.0002 (0.826)
**BMI**	−0.07 (0.342)	−0.05 (0.176)	−0.01 (0.545)	−0.01 (0.622)	0.01 (0.787)
**General quality of life**	1.17 (<0.001)	0.53 (<0.001)	0.22 (0.001)	0.38 (<0.001)	0.13 (0.165)

Source: elaborated by the authors. * Multiple linear regression. Values expressed in beta and *p* value. BMI: Body Mass Index.
